# MXene quantum dots for immunomodulation: redox activity and nano-strategies in inflammatory control and regenerative medicine

**DOI:** 10.1039/d6ra04639h

**Published:** 2026-07-06

**Authors:** Tareq Nayef AlRamadneh, Jasur Rizaev, Bakhtiyor Madiyorov, Thaer Abdul Allh, Faris Anad Muhammad, Jakhongir Norqulov, Neeraj Bainsal, Sharmin Smaeilpour

**Affiliations:** a Faculty of Allied Medical Sciences, Hourani Center for Applied Scientific Research, Al-Ahliyya Amman University Amman Jordan; b Doctor of Medical Sciences, Department of Public Health and Healthcare Management, Rector, Samarkand State Medical University Samarkand Uzbekistan; c Republican Specialized Scientific-Practical Medical Center of Oncology and Radiology Tashkent Uzbekistan; d Department of Oncology, Tashkent State Medical University Tashkent Uzbekistan; e Department of Tqnyat Altkhdyr, College Altqnyat Altbyh, The Islamic University Najaf Iraq; f College of Pharmacy, Alnoor University Nineveh Iraq; g Department of Medicine, Termez University of Economics and Service Termez Uzbekistan; h University Institute of Pharma Sciences, Chandigarh University Mohali Punjab India; i Young Researchers and Elite Club, Tehran Branch, Islamic Azad University Tehran Iran sharminsmaeilpour@gmail.com

## Abstract

MXene quantum dots (MQDs) have recently emerged as a distinct zero-dimensional derivative of MXenes, exhibiting unique physicochemical and electronic properties that extend their relevance beyond conventional nanomaterial applications. Owing to their ultrasmall size, rich surface termination chemistry, and transition metal-centered electronic structure, MQDs interact dynamically with biological systems and influence immune behavior in a context-dependent manner. This review provides the first comprehensive and focused overview of MQDs as immunomodulatory nanoplatforms for inflammatory control and regenerative medicine. We systematically analyze the structural evolution of MQDs from two-dimensional MXenes, highlighting how quantum confinement, surface chemistry, and redox-active electronic states define their nano–bio interface. Mechanistic insights into immune regulation are discussed through redox-sensitive signaling pathways, inflammasome dynamics, and immune cell reprogramming. Furthermore, experimental evidence demonstrating MQD-mediated modulation of T-cell activity, endothelial immunogenicity, and spatial immune organization in inflammatory, oncologic, and regenerative models is critically evaluated. By integrating materials chemistry, immunological mechanisms, and translational studies, this review outlines emerging nano-strategies for precision immune regulation. Finally, key challenges related to immunosafety, compositional engineering, and clinical translation are addressed, positioning MQDs as a promising and programmable class of immunoactive nanomaterials for future biomedical applications.

## Abstract 2

MXene quantum dots harness redox-driven immune reprogramming to modulate inflammation, promote regenerative responses, and advance precision nano-immunomodulation.

## Graphical abstract

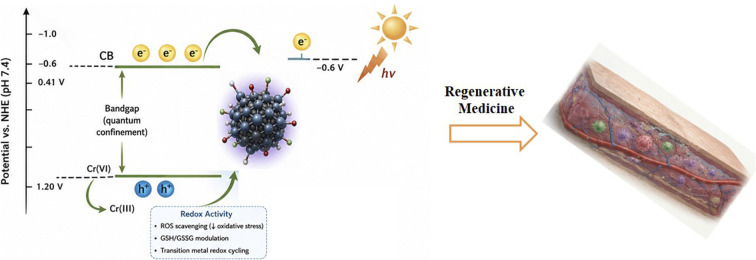

## 1. Introduction

The immune system plays a central role in maintaining tissue homeostasis, orchestrating inflammatory responses, and enabling regeneration following injury. While acute inflammasome is essential for host defense and tissue repair, dysregulated or chronic inflammatory responses contribute to a wide spectrum of pathological conditions, including autoimmune diseases, cancer, transplant rejection, and impaired tissue regeneration.^1–4^ In regenerative medicine, excessive immune activation remains a major barrier, often compromising the survival, integration, and function of transplanted cells or biomaterial scaffolds. Consequently, there is growing interest in developing strategies that do not simply suppress immunity, but rather recalibrate immune responses toward controlled inflammasome and tissue repair.^5–7^

Nanomaterials have emerged as powerful tools for immune modulation due to their tunable physicochemical properties and capacity to interact with immune cells at the molecular and cellular levels.^8,9^ Beyond acting as passive carriers for drugs or antigens, certain nanomaterials exhibit intrinsic immunological activity, influencing immune signaling, cell polarization, and cytokine secretion.^10,11^ This paradigm shift has given rise to the field of nano-immunomodulation, where material design itself becomes a determinant of immune outcome. However, achieving precise immune regulation requires nanoplatforms that combine structural tunability, biocompatibility, and predictable nano–bio interactions.^12–14^

Existing literature has extensively reviewed the immunomodulatory potential of various zero-dimensional platforms, such as carbon, graphene, and metallic quantum dots, as well as the broader biomedical applications of two-dimensional (2D) MXenes. However, these discussions often overlook the unique transition from 2D sheets to 0D quantum dots in the MXene family. While 2D MXenes are frequently studied for their physical reinforcement in scaffolds or photothermal properties, the specific biological signaling and intrinsic redox-mediated immune reprogramming triggered by their quantum dot derivatives (MQDs) remain poorly synthesized. A dedicated analysis is therefore required to distinguish the immunobiological profile of MQDs from both their bulk counterparts and other established quantum dot systems.

MXenes, a rapidly expanding family of two-dimensional transition metal carbides, nitrides, and carbonitrides, have attracted considerable attention for biomedical applications owing to their high surface area, electrical conductivity, and versatile surface chemistry.^15–17^ While early studies primarily focused on bulk or sheet-like MXenes, recent advances have enabled their transformation into zero-dimensional quantum dots, known as MXene quantum dots (MQDs). This dimensional downscaling introduces quantum confinement effects, increases surface atom exposure, and fundamentally alters electronic and interfacial properties. As a result, MQDs cannot be regarded merely as fragmented MXene sheets but rather as a distinct nanomaterial class with unique biological implications.^18,19^

MQDs exhibit several features that are particularly relevant for immune regulation. Their ultrasmall size (typically <10 nm) facilitates rapid diffusion, cellular uptake, and access to intracellular compartments. Rich surface terminations inherited from MXene chemistry confer hydrophilicity, colloidal stability, and dynamic surface charge behavior in physiological environments.^20,21^ Importantly, the presence of transition metal centers endows MQDs with redox-active electronic states capable of participating in electron transfer and reactive oxygen species (ROS)-related processes. These attributes collectively position MQDs at the intersection of materials chemistry and immune signaling.^22,23^

Redox processes are increasingly recognized as central regulators of immune function. ROS species act not only as cytotoxic agents but also as second messengers that shape immune cell activation, differentiation, and fate decisions. Redox-sensitive pathways regulate macrophage polarization, T-cell activation thresholds, inflammasome assembly, and antigen-presenting cell maturation. Subtle modulation of intracellular redox tone can therefore shift immune responses from pathological inflammasome toward resolution and regeneration. Nanomaterials with controllable redox activity offer an opportunity to engage these pathways in a programmable manner.^24–27^ In this context, MQDs represent a particularly intriguing platform, as their redox behavior arises intrinsically from their electronic structure rather than from exogenous payloads.

Hybridization strategies are increasingly viewed as a practical route to amplify the immunomodulatory performance of MQDs and overcome limitations of pristine dots in complex *in vivo* settings. By integrating MQDs with complementary matrices—such as injectable hydrogels, biodegradable polymers, liposomes, or porous frameworks (*e.g.*, MOFs/COFs)—it becomes possible to improve colloidal stability, prolong local retention, and reduce off-target distribution. Importantly, hybrid constructs can enable spatiotemporal control over redox signaling and immune activation by combining MQDs with responsive components (pH-, enzyme-, or light-triggered systems).^19–21^ Such composites may also support multimodal therapy (*e.g.*, immunomodulation coupled with photothermal/photodynamic effects) and provide bioactive microenvironments that coordinate immune resolution with tissue regeneration.

Despite the rapid emergence of MQDs in biomedical research, their immunomodulatory roles have remained fragmented across studies focused on specific disease models or material compositions. Experimental evidence now demonstrates that MQDs can selectively attenuate pro-inflammatory T-cell responses, promote regulatory immune phenotypes, modulate endothelial immunogenicity, reshape tumor immune microenvironments, and integrate seamlessly within regenerative scaffolds without impairing stem cell viability.^28–30^ These findings suggest that MQDs operate as active immuno-instructive materials rather than inert nanocomponents. However, a unified framework connecting MQD physicochemical identity, redox activity, immune mechanisms, and translational performance has been lacking.

To bridge this conceptual divide, the present review provides the first comprehensive and focused synthesis of MQDs as specialized immunomodulatory nanoplatforms for inflammatory control and regenerative medicine. Rather than surveying MXenes broadly or duplicating existing reviews on generic quantum dots, this article concentrates specifically on the unique bio-interfacial mechanisms of MQDs and their zero-dimensional attributes. We begin by examining the structural evolution and surface chemistry that distinguish MQDs from their two-dimensional counterparts, emphasizing how quantum confinement and surface terminations define nano–bio interfaces. We then discuss molecular and cellular mechanisms of immune modulation, with particular attention to redox-sensitive signaling, inflammasome dynamics, and immune cell reprogramming. Subsequently, experimental and translational studies are critically analyzed to illustrate how MQDs perform in inflammatory, oncologic, and regenerative contexts. Finally, emerging design principles, immunosafety considerations, and future directions are outlined to guide the rational engineering of next-generation immunomodulatory MQDs. By integrating materials science, immunology, and translational research, this review aims to establish a conceptual foundation for precision nano-immunomodulation using MQDs. Such an approach may enable the development of advanced therapies that balance immune control with regenerative efficacy, addressing longstanding challenges at the interface of inflammasome and tissue repair.

**Fig. 1:**
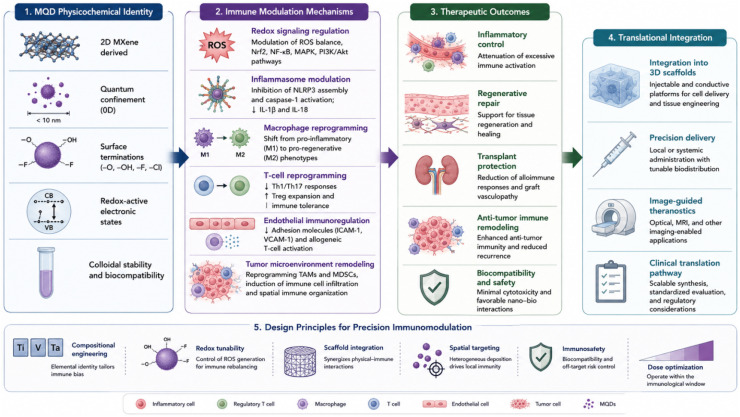
Conceptual framework illustrating the physicochemical properties, immunomodulatory mechanisms, therapeutic applications, and translational design principles of MQDs in inflammatory regulation and regenerative immunomedicine.

The Fig. 1 summarizes the conceptual framework of the present review and illustrates how MQDs function as programmable immunomodulatory nanoplatforms. The figure highlights the relationship between MQD physicochemical characteristics, including quantum confinement, surface terminations, and redox-active properties, and their downstream immunological effects. MQDs regulate multiple immune pathways through redox signaling, inflammasome modulation, macrophage polarization, T-cell reprogramming, and tumor microenvironment remodeling. These mechanisms collectively contribute to therapeutic outcomes in inflammatory control, regenerative medicine, transplantation, and cancer immunotherapy. The scheme further outlines key design principles required for precision nano-immunomodulation and future clinical translation of MQD-based platforms.

## 2. MQDs as an emerging 0D immunoactive nanoplatform: structural evolution, surface chemistry, and distinctive physicochemical identity

### 2.1. Structural downscaling from 2D MXenes to 0D quantum dots: dimensional transition and confinement effects

MQDs represent the zero-dimensional (0D) evolution of the parent two-dimensional (2D) MXene family, derived from layered transition metal carbides, nitrides, or carbonitrides (M_*n*+1_X_*n*_T_*x*_). The transition from extended 2D sheets to ultrasmall nanodomains—typically below 10 nm—induces profound structural and electronic reorganization beyond mere size reduction. This dimensional downscaling introduces quantum confinement effects, alters edge-to-basal plane ratios, and significantly increases surface atom exposure, thereby redefining the physicochemical identity of the material.^31,32^

In 2D MXenes, electron transport is predominantly governed by in-plane metallic conductivity facilitated by delocalized d-electrons of transition metals such as Ti, V, or Ta. Upon fragmentation into MQDs, spatial confinement modifies electronic density distribution, often leading to partial bandgap opening, localized states, and enhanced photoluminescent behavior. The shift from metallic sheets to quasi-semiconducting nanodomains introduces discrete energy levels and size-dependent optical responses, which distinguish MQDs fundamentally from their bulk counterparts.^33,34^

Moreover, the edge-to-volume ratio dramatically increases in MQDs. Edge atoms, characterized by unsaturated coordination environments, exhibit higher chemical reactivity compared to basal-plane atoms. This structural reconfiguration amplifies catalytic potential, redox responsiveness, and interaction probability with surrounding biological media. Unlike graphene or carbon quantum dots, MQDs retain transition-metal-centered electronic features, enabling d-orbital participation in charge transfer processes.

Crystallographically, MQDs maintain the layered heritage of MXenes but often display lattice distortions and increased defect density due to top-down exfoliation or hydrothermal fragmentation routes. These structural imperfections are not merely synthetic artifacts; they actively contribute to electronic heterogeneity and reactive site availability.^35–38^ Importantly, defect engineering at the nanoscale can modulate electron localization and surface coordination chemistry. Thus, the 2D-to-0D transition in MXenes is not a trivial miniaturization process. It redefines electronic structure, defect landscape, catalytic potential, and interfacial reactivity. MQDs should therefore be conceptualized as a distinct nanomaterial class rather than reduced fragments of MXene sheets.

**Fig. 2:**
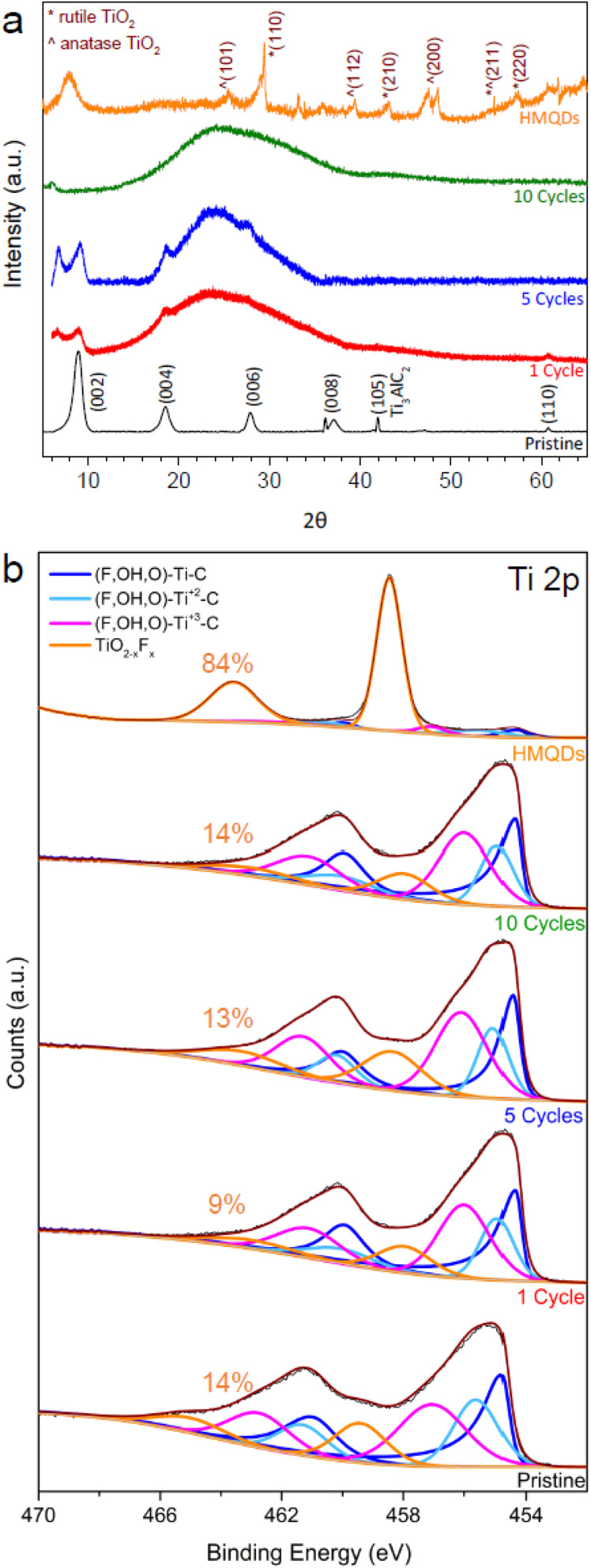
(a) XRD patterns showing structural evolution from multilayer MXene to MQDs; (b) Ti 2p XPS spectra illustrating chemical-state redistribution during dimensional downscaling. Adapted with permission from ref. 38. © 2021 American Chemical Society.

Panel (a) in Fig. 2 presents the XRD evolution of pristine multilayer Ti_3_C_2_T_*x*_ MXene, delaminated nanosheets, and MQDs generated through successive nebulization cycles.^38^ The progressive shift of the (002) diffraction peak toward lower angles, accompanied by peak broadening and intensity attenuation, reflects a systematic increase in interlayer spacing and loss of long-range crystallographic order. These changes indicate the disruption of stacked lamellar domains and the emergence of nanoscale confinement rather than simple exfoliation. The gradual disappearance of higher-order reflections further suggests reduced interlayer coherence and increased structural heterogeneity, consistent with the transition from extended 2D frameworks to spatially confined 0D nanodomains.

Panel (b) shows high-resolution Ti 2p XPS spectra, revealing pronounced changes in titanium chemical states during structural downscaling. The relative increase of TiO_2−*x*_F_*x*_ species in MQDs compared to pristine MXene indicates enhanced surface oxidation and coordination disorder induced by dimensional confinement. These variations are not merely surface artifacts but reflect altered electronic environments arising from reduced coordination numbers and defect-rich structures. Such chemical-state redistribution directly accompanies the structural transformation, reinforcing that MQDs possess a redefined crystallographic and electronic identity rather than being fragmented MXene residues.

### 2.2. Surface termination chemistry and functional group dynamics in biological media

A defining characteristic of MXene-derived nanostructures is their rich surface termination profile. During synthesis—commonly *via* selective etching of MAX phases—surface terminations such as –O, –OH, and –F are introduced. In MQDs, due to their extremely high surface-to-volume ratio, these terminations dominate physicochemical behavior. Unlike carbon-based quantum dots whose surfaces are largely governed by sp^2^/sp^3^ carbon hybridization, MQDs exhibit metal–oxygen and metal–hydroxyl coordination environments. These terminations regulate hydrophilicity, colloidal stability, zeta potential, and coordination affinity toward ions or biomolecules. The relative abundance of –O *versus* –OH groups influences hydrogen bonding capacity and proton exchange behavior, while residual –F groups may alter electrostatic interactions and surface polarity.^39–41^

In aqueous environments, dynamic surface reconstruction may occur. Protonation–deprotonation equilibria alter surface charge density depending on pH, influencing dispersion stability and electrostatic interaction potential. Additionally, ligand exchange processes can replace original terminations with biomolecule-derived moieties, especially in protein-rich environments. The metal centers (*e.g.*, Ti^3+^/Ti^4+^ redox states) further contribute to surface chemistry complexity. Mixed-valence states enable redox-active interfaces capable of participating in electron transfer processes. Importantly, oxidation susceptibility can lead to gradual transformation into metal oxides or oxyhydroxides, modifying surface energetics and catalytic activity over time. Surface functionalization strategies—such as amination, PEGylation, or heteroatom doping (N, S, B)—introduce further chemical diversity.^43–46^ These modifications alter electron density distribution and coordination chemistry without fundamentally changing the MXene-derived backbone. From a materials chemistry perspective, MQDs present a uniquely tunable surface chemistry platform combining metallic character with quantum-scale reactivity.^42^

**Fig. 3:**
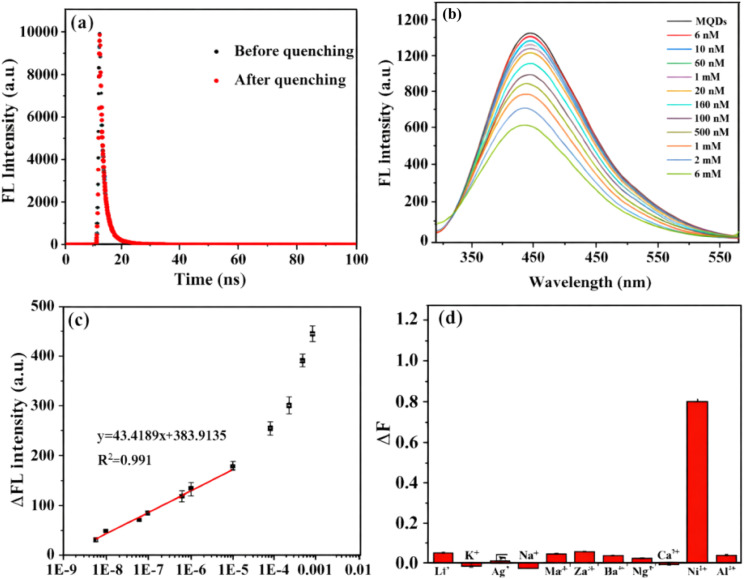
(a) FL lifetime decay; (b) concentration-dependent FL quenching; (c) Stern–Volmer calibration; (d) ion selectivity response of MQDs. Adapted with permission from ref. 45. © 2021 American Chemical Society.

Panels (a) and (b) in Fig. 3 illustrate the fluorescence lifetime and intensity response of MQDs upon Ni^2+^ coordination, highlighting the dominant role of surface functional groups.^45^ The minimal change in fluorescence lifetime despite strong emission quenching indicates a surface-mediated interaction rather than bulk electronic disruption. Amino and oxygen-containing terminations act as coordination sites for Ni^2+^, enabling nonradiative energy dissipation through interfacial electron transfer. This behavior underscores how surface chemistry, amplified by the high surface-to-volume ratio of MQDs, governs their optical response in solution.

Panels (c) and (d) further demonstrate the chemical selectivity imposed by surface terminations. The linear Stern–Volmer relationship and negligible interference from competing metal ions confirm that fluorescence modulation originates from specific coordination interactions rather than nonspecific electrostatic effects. Such selective quenching reflects dynamic surface reconstruction and ligand–metal affinity under aqueous conditions. These results emphasize that MQD surface terminations function as chemically active interfaces, dynamically regulating charge transfer and interaction specificity in complex chemical environments.

### 2.3. Electronic structure, redox behavior, and catalytic reactivity

The electronic architecture of MQDs is governed by transition-metal d orbitals interacting with carbon or nitrogen p orbitals within confined domains. Quantum confinement introduces discrete electronic states, shifting optical absorption edges and enabling photoluminescence—features not prominent in bulk MXenes. A critical distinction of MQDs lies in their redox versatility. The presence of variable oxidation states (*e.g.*, Ti^3+^/Ti^4+^) facilitates electron donation and acceptance processes. These redox-active sites enable catalytic transformations, including peroxide decomposition and radical generation under specific conditions. Unlike inert carbon QDs, MQDs exhibit intrinsic catalytic behavior rooted in transition-metal chemistry.^47,48^

Charge transfer processes in MQDs are influenced by surface terminations and defect density. Oxygen vacancies and lattice distortions create localized electronic states that enhance electron–hole separation efficiency. This contributes to their photothermal and photocatalytic properties, especially under UV or NIR irradiation. Furthermore, MQDs display high extinction coefficients and strong broadband absorption, attributed to d–d transitions and surface plasmon-like behavior in certain compositions. These optical-electronic characteristics enable energy conversion processes distinct from classical semiconductor quantum dots.

Electrochemically, MQDs demonstrate efficient charge mobility and low interfacial resistance when integrated into conductive systems. Their nanoscale dimensions facilitate rapid electron exchange kinetics, making them suitable for redox-driven applications.^49,50^ Importantly, electronic tunability *via* doping or compositional variation allows tailoring of band structure and catalytic thresholds. Thus, MQDs occupy an unusual materials space between metallic nanoclusters and semiconductor quantum dots, combining quantum confinement with transition-metal-centered redox chemistry.

**Fig. 4:**
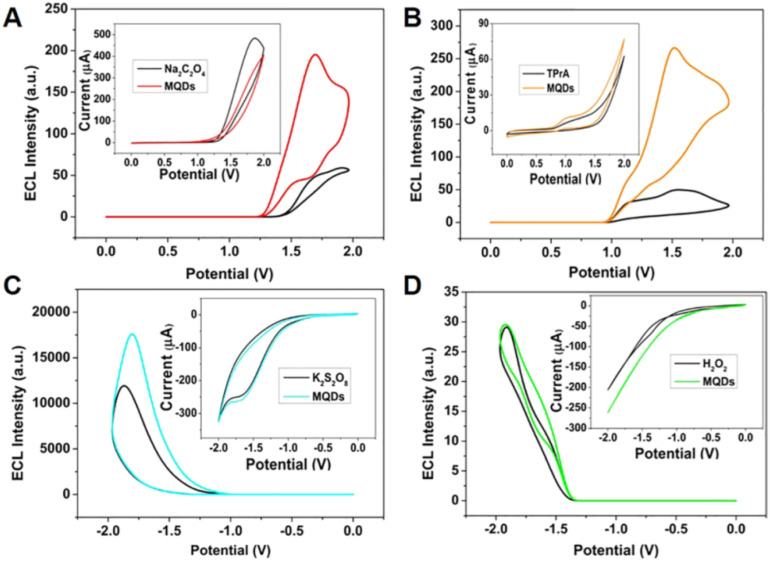
(A) ECL response of MQDs with sodium oxalate *via* oxidation-driven radical formation. (B) Tripropylamine-assisted anodic excitation of MQDs through electron transfer. (C) Strong ECL signal with potassium persulfate due to efficient sulfate radical redox coupling. (D) Negligible ECL activity with hydrogen peroxide owing to poor energetic matching. Adapted with permission from ref. 50. © 2021 American Chemical Society.

Fig. 4 systematically illustrates the electrochemiluminescence (ECL) behavior of MQDs in the presence of different coreactants, directly reflecting their transition-metal-centered electronic structure and redox activity.^50^ Panels (A) and (B) demonstrate that sodium oxalate and tripropylamine induce ECL responses through oxidation-driven electron transfer pathways, confirming that MQDs can access excited electronic states *via* both anodic and cathodic processes. These responses arise from the participation of confined d-orbital states that facilitate reversible electron exchange under electrical stimulation.

Panel (C) exhibits a markedly enhanced ECL signal in the potassium persulfate system, accompanied by a negative shift in the onset potential. This behavior indicates efficient generation of sulfate radical intermediates that interact strongly with reduced MQDs, promoting the formation of excited MQD* states. Such efficient redox coupling highlights the ability of MQDs to stabilize transient charge-separated states, a feature enabled by quantum confinement and mixed-valence metal centers.

In contrast, panel (D) shows negligible ECL activity with hydrogen peroxide, emphasizing that effective luminescence requires energetic compatibility between MQD electronic states and redox partners. Collectively, the panel-resolved data confirm that MQDs exhibit intrinsic electrochemical activity governed by confined electronic states and redox-flexible metal centers, positioning them as catalytically active quantum nanomaterials rather than passive fluorescent emitters.

### 2.4. Nano–bio interface formation: colloidal stability, protein corona, and interfacial thermodynamics

When introduced into biological environments, MQDs rapidly establish a nano–bio interface governed by physicochemical parameters rather than biological intent. Their ultrasmall size facilitates high diffusivity and extensive surface exposure, making interfacial phenomena dominant determinants of behavior. Colloidal stability is primarily influenced by electrostatic repulsion and hydration shell formation. Surface terminations confer negative charge under physiological pH, promoting dispersion stability in aqueous systems. However, ionic strength and divalent cations may screen surface charge, inducing aggregation. Aggregation state directly alters effective surface area and diffusion dynamics.^51,52^

Protein corona formation occurs almost instantaneously in serum-containing media. Adsorption is driven by electrostatic attraction, hydrogen bonding, van der Waals forces, and coordination interactions between metal centers and protein residues. The corona composition is dynamic and depends on particle size, surface chemistry, and environmental conditions. Importantly, corona formation modifies hydrodynamic diameter, surface charge, and interfacial free energy. Thermodynamically, the nano–bio interface seeks minimization of interfacial energy. High surface energy in MQDs favors adsorption phenomena. Competitive binding between biomolecules reshapes the particle's outer identity, effectively redefining its biological presentation.^52–54^

**Fig. 5:**
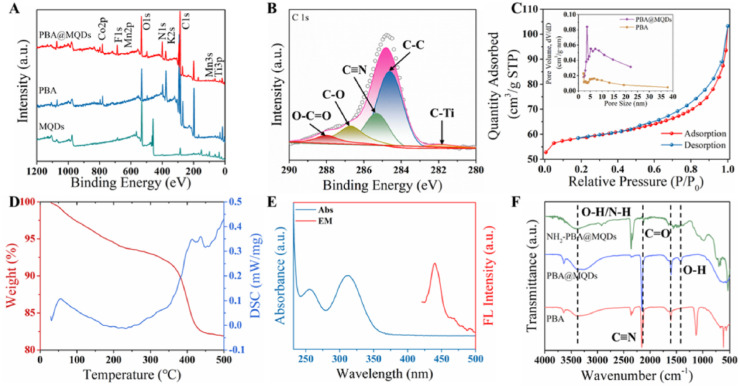
Physicochemical and interfacial characterization of PBA@MQDs. (A) Comparative XPS survey spectra of MQDs, PBA, and PBA@MQDs. (B) High-resolution C 1s XPS spectrum of PBA@MQDs. (C) N_2_ adsorption–desorption isotherms (inset: pore size distribution) confirming the mesoporous structure. (D) TGA curves illustrating the enhanced thermal stability of the composite. (E) UV-vis absorption and PL spectra (*λ*_ex_ = 350 nm). (F) FT-IR spectra showing the successful synthesis and subsequent surface amination (NH_2_-PBA@MQDs). Reproduced with permission from ref. 51. © 2025 Elsevier B.V.

The structural integrity and surface chemistry of the synthesized PBA@MQDs were rigorously characterized to evaluate their potential behavior at the nano–bio interface. X-ray photoelectron spectroscopy (XPS) analysis (Fig. 5A) confirms the successful integration of MQDs with the PBA framework, where the presence of Co and Mn peaks alongside the C 1s signals indicates a stable coordination self-assembly. Specifically, the C 1s spectrum (Fig. 5B) reveals distinct peaks for C–Ti, C≡N, and oxygen-containing groups (C–O and O–C=O), which are pivotal in governing the interfacial free energy and the subsequent protein corona formation. The high Brunauer–Emmett–Teller (BET) surface area of 183.7 m^2^ g^−1^, derived from the type-IV N_2_ adsorption–desorption isotherms (Fig. 5C), suggests a highly active mesoporous architecture that enhances molecular diffusion and provides extensive sites for biomolecular adsorption. Thermal stability, a critical factor for maintaining colloidal identity in fluctuating environments, was verified *via* thermogravimetric analysis (TGA). As shown in Fig. 5D, the PBA@MQDs exhibit significantly higher thermal resistance compared to pristine MQDs, which is attributed to the PBA-doping that stabilizes the organic–inorganic interface. Optical characterization (Fig. 5E) highlights the characteristic absorption peaks and a robust photoluminescence emission at 440 nm, facilitating real-time monitoring of the nano–bio interaction. Finally, the Fourier-transform infrared (FT-IR) spectra (Fig. 5F) confirm the successful amino-functionalization (NH_2_-stretching at 3410 cm^−1^), a modification that redefines the surface charge and coordination capacity of the particles, ultimately dictating their biological identity and interaction with target aptamers.

Beyond confirming successful synthesis, the physicochemical features of PBA@MQDs establish a clear structure–property relationship governing their nano–bio behavior. The coexistence of oxygen-containing functional groups, high specific surface area, mesoporous architecture, and enhanced interfacial stability collectively promotes efficient biomolecular adsorption, charge-transfer processes, and colloidal persistence in biological media. Similar to recent heterojunction-based nanomaterials, where interfacial electronic structures dictate functional performance,^86^ the engineered surface chemistry and coordination environment of PBA@MQDs are expected to regulate protein-corona evolution, aptamer recognition, and downstream immunomodulatory interactions.

### 2.5. Synthesis and functional modification strategies

The structural and electronic diversity of MQDs is intrinsically tied to their synthetic lineage. Top-down fabrication, the most prevalent method, involves the fragmentation of bulk, multilayered MXene precursors (such as Ti_3_C_2_T_*x*_) through chemical etching, or mechanical processes like hydrothermal, electrochemical, or ultrasonic exfoliation. This approach effectively exploits the intrinsic layered structure, yielding nanostructures that retain the transition-metal-centered electronic features of the parent phase, albeit often introducing structural defects. Conversely, bottom-up assembly—typically involving solvothermal synthesis from metal and carbon precursors—enables greater command over particle size, morphology, and purity. While often more complex, bottom-up methods offer a precise pathway to minimize structural artifacts and achieve highly homogeneous nanostructures with predictable crystallographic properties.^32–34^

Beyond conventional HF-assisted etching routes, increasing attention has been directed toward fluorine-free and environmentally benign synthesis strategies to improve the scalability and biosafety of MQD production. Alternative approaches based on electrochemical exfoliation, molten-salt etching, hydrothermal conversion, and other fluoride-free processes can minimize hazardous waste generation while preserving the structural and electronic characteristics of the resulting nanodots. Such green manufacturing routes are particularly attractive for biomedical applications because they reduce residual fluorinated surface species and facilitate regulatory compliance for large-scale production. Recent studies have highlighted the feasibility of scalable HF-free processing pathways for advanced MXene-derived nanostructures.^90^

Post-synthetic functionalization is the critical bridge translating raw MQDs into sophisticated bio-instructive agents. Functionalization strategies generally bifurcate into covalent and non-covalent modalities. Covalent grafting—utilizing surface hydroxyl or oxygen groups—enables the attachment of biocompatible polymers like PEG, or targeting ligands, which significantly dictates colloidal stability and minimizes non-specific protein corona formation, as detailed in Section 2.4. Furthermore, heteroatom doping (*e.g.*, N, S, B, or P integration) during or after synthesis serves to tune the electronic density and bandgap, thereby optimizing the redox activity described in Section 2.3. On the other hand, non-covalent strategies, such as the electrostatic adsorption of surfactants or biomolecules, offer a reversible and dynamic mechanism to modify the MQD–bio interface. These methods permit the rapid assembly of hybrid materials without altering the intrinsic metal-carbide backbone.^38–40^ By strategically combining synthetic control with surface-engineering techniques, researchers can manipulate the surface termination landscape, mitigating aggregation while enhancing catalytic and optical responses. Ultimately, these strategies empower the engineering of “intelligent” MQDs, transforming them from passive nano-fragments into highly stable, targeted, and responsive platforms capable of navigating complex biological environments for precise immunomodulation.

Table 1 distills the key physicochemical parameters that define MQDs as a distinct nano-immunoactive material class. Rather than reiterating structural descriptions, it emphasizes how quantum confinement, surface termination density, redox-active electronic states, and interfacial thermodynamics collectively shape nano–bio interactions. These attributes govern protein corona formation, colloidal stability, and oxidative responsiveness—critical determinants of downstream immune engagement. The comparison with 2D MXenes highlights that immunological relevance emerges specifically from the 0D transformation rather than the parent material itself.

**Table 1:** Physicochemical attributes of MQDs relevant to immunoactive nano–bio interfaces

Attribute category	MQD feature	Origin	Physicochemical implication	Nano–bio interface impact	Distinction from 2D MXenes
Dimensionality	0D confinement	Top-down fragmentation	Discrete energy states	Enhanced molecular interactions	Loss of long-range conductivity
Size regime	<10 nm	Quantum downscaling	High surface atom fraction	Rapid diffusion, cellular access	Sheet-to-dot transition
Surface terminations	–O, –OH, –F	Etching chemistry	Hydrophilicity, charge tunability	Protein adsorption control	Higher termination density
Electronic structure	d-orbital dominated	Transition metal core	Redox versatility	ROS-responsive interfaces	Partial bandgap opening
Defect density	Vacancies, distortions	Hydrothermal cleavage	Localized electronic states	Reactive coordination sites	Defect-amplified behavior
Colloidal behavior	Stable dispersions	Surface charge hydration	Low aggregation tendency	Predictable bio-distribution	Superior to bulk MXenes
Oxidation dynamics	Partial metal oxidation	Aqueous exposure	Surface reconstruction	Time-dependent bioactivity	Faster than 2D sheets
Optical response	PL & broadband absorption	Quantum confinement	Energy conversion	Tracking & stimulation	Absent in bulk MXenes
Interfacial thermodynamics	High surface energy	Nanoscale curvature	Adsorption-driven interactions	Dynamic corona formation	Enhanced interface dominance

## 3. Molecular and cellular mechanisms of immune modulation by MQDs: redox signaling, inflammasome dynamics, and immune cell reprogramming

### 3.1. Redox-driven immune signaling and oxidative checkpoint modulation

ROS function not merely as cytotoxic byproducts but as central second messengers in immune signaling networks. Immune cells—including macrophages, dendritic cells, neutrophils, and T lymphocytes—rely on tightly regulated redox gradients to orchestrate activation, differentiation, and effector responses. Perturbation of intracellular redox balance can therefore reshape immune outcomes through modulation of redox-sensitive transcription factors and kinase cascades. ROS-sensitive signaling nodes such as NF-κB, MAPKs (ERK, JNK, p38), PI3K/Akt, and Nrf2 operate as molecular switches that translate oxidative fluctuations into transcriptional programs. Moderate ROS elevation can enhance pro-inflammatory gene transcription *via* IκB degradation and NF-κB nuclear translocation. Conversely, activation of the Nrf2-Keap1 axis promotes antioxidant gene expression (HO-1, NQO1, GCLC), shifting cells toward cytoprotective and anti-inflammatory states.^55–57^

Redox modulation also influences T cell receptor (TCR) signaling thresholds. Oxidation of cysteine residues within phosphatases such as SHP-1 and PTEN alters their catalytic activity, thereby modifying downstream phosphorylation events. This redox checkpoint determines whether T cells undergo activation, anergy, or apoptosis. Similarly, macrophage polarization states (classically activated M1 *versus* alternatively activated M2 phenotypes) are tightly linked to metabolic-redox coupling, with glycolysis-associated ROS favoring inflammatory phenotypes and mitochondrial oxidative metabolism supporting reparative programs. Importantly, redox perturbations influence antigen-presenting cell maturation. Oxidative signals regulate co-stimulatory molecule expression (CD80/CD86) and cytokine secretion profiles (IL-12, IL-10), thereby shaping adaptive immune polarization. Sustained oxidative stress, however, may induce immunosuppressive circuits *via* induction of heme oxygenase-1 or activation of tolerogenic dendritic phenotypes.^58^ Thus, modulation of cellular redox tone acts as a master regulatory axis governing innate and adaptive immune balance. The immune consequences of redox-active nanomaterials are therefore determined not by ROS generation alone, but by magnitude, duration, subcellular localization, and the cellular antioxidant buffering capacity.

Mechanistically, MQDs can enable directional redox regulation by leveraging interfacial electronic structures that govern charge separation and electron flux. Analogous to S-scheme heterojunctions where built-in electric fields promote efficient carrier migration and steer radical pathways, engineered MQD interfaces (*via* surface terminations/heterojunction-like coupling) may bias electron–hole utilization toward selective ROS quenching or generation in specific microdomains.^59^ Likewise, defect- and vacancy-associated charge transfer (*e.g.*, oxygen-vacancy-driven electron redistribution) provides a model for how MQD defects could tune O_2_ adsorption/electron donation and thereby reshape immune redox checkpoints with higher specificity.^60^

**Fig. 6:**
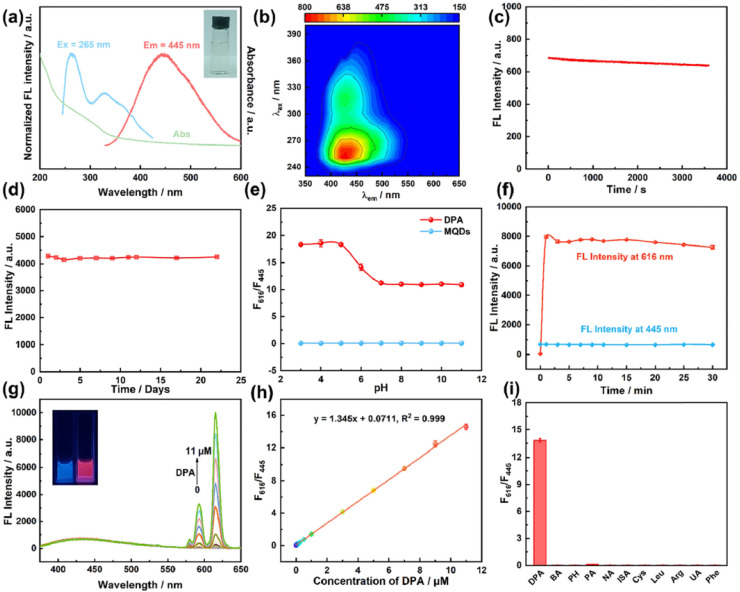
Optical properties and sensing performance of MQD-based fluorescent probes. (a and b) UV-vis absorption and photoluminescence spectra. (c and d) Photostability and storage stability. (e and f) pH-dependent fluorescence response and sensing kinetics. (g and h) Ratiometric fluorescence response and calibration curve toward DPA. (i) Selectivity analysis against potential interferents. Reproduced from ref. 56 under CC BY-NC 3.0. © 2024 Royal Society of Chemistry.

To further illustrate the analytical capabilities of MQD-based platforms relevant to immune-microenvironment studies, Fig. 6 summarizes their optical and sensing properties. As shown in Fig. 6a and b, MQDs exhibit broad UV-vis absorption and stable photoluminescence emission, providing favorable optical characteristics for fluorescence-based biological monitoring. The excellent photostability and storage stability observed in Fig. 6c and d indicate that the optical signal remains reliable during prolonged experimental investigations. Furthermore, the pH-dependent fluorescence behavior and rapid sensing kinetics presented in Fig. 6e and f demonstrate the applicability of MQDs across diverse intracellular environments, including endosomal and lysosomal compartments where immune signaling events frequently occur. The ratiometric fluorescence response and calibration performance shown in Fig. 6g and h, together with the selectivity evaluation in Fig. 6i, confirm the sensitivity and robustness of the sensing platform. Although the reported system was originally developed for DPA detection, the results collectively highlight the broader potential of MQD-based fluorescent probes for monitoring biochemical and metabolic changes associated with redox regulation, thereby supporting future investigations of immune signaling and microenvironmental remodeling. It should be noted that the DPA-sensing system presented in Fig. 6 is not intended as direct evidence of MQD-mediated immunomodulation. Rather, it serves as a representative example of how MQD-based optical platforms can be adapted for monitoring biochemical and metabolic signals within immune microenvironments, thereby providing analytical tools to investigate redox-dependent immune regulation.

### 3.2. Inflammasome regulation and pyroptotic signaling cascades

The inflammasome represents a cytosolic multiprotein platform responsible for sensing danger-associated molecular patterns (DAMPs) and pathogen-associated molecular patterns (PAMPs). Among these complexes, the NLRP3 inflammasome is the most extensively characterized and acts as a convergence node for metabolic stress, ionic imbalance, mitochondrial dysfunction, and lysosomal perturbation. Inflammasome activation proceeds through a two-signal model. The priming phase involves NF-κB-mediated upregulation of NLRP3 and pro-IL-1β/pro-IL-18 transcription. The activation phase is triggered by potassium efflux, mitochondrial ROS accumulation, cathepsin release, or ATP-mediated purinergic signaling. Assembly of the NLRP3-ASC-caspase-1 complex results in caspase-1 activation, cleavage of pro-inflammatory cytokines, and gasdermin D-mediated membrane pore formation, culminating in pyroptosis.^61–63^

Regulation of inflammasome dynamics is highly sensitive to intracellular stress signatures. Mitochondrial ROS amplify NLRP3 activation, whereas autophagy-mediated clearance of damaged mitochondria suppresses inflammasome assembly. Additionally, post-translational modifications—including ubiquitination, phosphorylation, and S-nitrosylation—modulate inflammasome stability and activity. Beyond macrophages, inflammasome components are expressed in endothelial cells, epithelial cells, and certain lymphocyte subsets, implicating this pathway in systemic inflammatory disorders, graft rejection, and chronic degenerative diseases. Controlled attenuation of inflammasome activation may therefore reduce IL-1β-driven inflammasome and tissue damage. Conversely, excessive suppression may impair host defense.^64–66^

Importantly, pyroptosis differs fundamentally from apoptosis and necrosis. It is a lytic, inflammatory form of programmed cell death characterized by cytokine release and amplification of immune recruitment. The extent and context of inflammasome modulation determine whether immune responses resolve inflammasome or escalate tissue injury. Thus, inflammasome signaling constitutes a discrete mechanistic axis of immune modulation, governed by cytosolic stress integration rather than surface receptor signaling alone.

### 3.3. Immune cell reprogramming: macrophage plasticity and T cell fate decisions

Immune responses are not binary but exist along dynamic phenotypic continua. Macrophages exhibit remarkable plasticity, transitioning between pro-inflammatory (M1-like) and pro-regenerative (M2-like) states in response to microenvironmental cues. This polarization spectrum is orchestrated by cytokine signaling (IFN-γ, IL-4, IL-13), metabolic rewiring, and transcriptional regulators such as STAT1, STAT6, IRF5, and PPARγ. M1 macrophages rely predominantly on glycolysis and produce nitric oxide, IL-6, TNF-α, and IL-1β, contributing to pathogen clearance and acute inflammasome. In contrast, M2 macrophages favor oxidative phosphorylation and fatty acid oxidation, secreting IL-10, TGF-β, and growth factors that support tissue remodeling and angiogenesis. Shifts in metabolic flux alter epigenetic landscapes through changes in acetyl-CoA, succinate, and α-ketoglutarate levels, thereby stabilizing polarization states.^67,68^

Adaptive immunity is equally sensitive to microenvironmental signals. CD4^+^ T cells differentiate into Th1, Th2, Th17, or regulatory T (Treg) subsets depending on cytokine milieu and antigenic context. T cell fate decisions are regulated by lineage-defining transcription factors such as T-bet, GATA3, RORγt, and FoxP3. Perturbations in antigen presentation strength, co-stimulation intensity, and metabolic status influence clonal expansion *versus* tolerance induction. Importantly, immune reprogramming is reversible. Epigenetic remodeling, metabolic checkpoint regulation (mTOR, AMPK), and cytokine feedback loops enable dynamic adaptation rather than fixed differentiation. Controlled modulation of these pathways can either dampen chronic inflammasome or enhance protective immunity.^69,70^ Thus, immune cell reprogramming represents a systems-level mechanism in which metabolic, transcriptional, and cytokine networks converge to determine inflammatory *versus* regenerative outcomes.

### 3.4. Microenvironmental remodeling and immunological crosstalk

Immune responses are embedded within complex tissue microenvironments composed of stromal cells, extracellular matrix (ECM), vascular networks, and soluble mediators. Modulation of immune activity therefore extends beyond individual cell signaling to encompass spatial and biochemical reorganization of the local milieu. Cytokine gradients govern immune cell trafficking and retention. Chemokines such as CCL2, CXCL8, and CXCL10 orchestrate recruitment of monocytes, neutrophils, and T cells. Alterations in chemokine expression reshape immune infiltration patterns and dictate inflammatory persistence *versus* resolution.^71,72^

Extracellular matrix components—including collagen, fibronectin, and hyaluronic acid—serve not only structural roles but also biochemical signaling functions. ECM stiffness influences macrophage phenotype *via* mechanotransduction pathways involving integrins, focal adhesion kinase (FAK), and YAP/TAZ signaling. Mechanical cues can therefore bias inflammatory *versus* reparative programs independent of cytokine signaling. Endothelial activation represents another critical interface. Upregulation of adhesion molecules (ICAM-1, VCAM-1) enhances leukocyte extravasation. Modulation of endothelial inflammatory tone directly impacts immune cell entry into tissues.^73–75^

Additionally, intercellular communication through extracellular vesicles and danger signals amplifies immune cascades. Release of ATP, HMGB1, and mitochondrial DNA reinforces inflammatory loops, whereas anti-inflammatory mediators promote resolution. Microenvironmental remodeling ultimately determines whether immune activation leads to tissue destruction or coordinated repair. Therefore, immune modulation must be interpreted within the spatial and mechanical context of tissue-level organization rather than isolated intracellular pathways.^76–78^

### 3.5. Epigenetic rewiring and metabolic-epigenetic coupling in MQD-mediated immunomodulation

Beyond transient kinase phosphorylation and transcriptional activation, the long-term immunomodulatory effects of MQDs are increasingly linked to their capacity to induce epigenetic rewiring. Epigenetic modifications—such as DNA methylation, histone acetylation, and chromatin remodeling—act as stable molecular memories that lock immune cells into specific functional phenotypes. Crucially, these epigenetic processes are fundamentally coupled to cellular metabolism, as key metabolic intermediates serve as essential cofactors or substrates for chromatin-modifying enzymes. For instance, acetyl-CoA is the sole donor for histone acetyltransferases (HATs), while α-ketoglutarate (α-KG) and NAD^+^ act as vital co-substrates for JmjC-domain-containing histone demethylases and sirtuins, respectively. By entering cells *via* active endocytic pathways, MQDs can selectively reprogram these metabolic-epigenetic circuits.

Specifically, the redox-active nature and surface transition metal centers of MQDs (such as Ti or Ta) can modulate mitochondrial respiratory chain kinetics and glycolysis rates. This metabolic perturbation alters the intracellular ratios of key metabolites like succinate, fumarate, and α-KG. Accumulation of fumarate or succinate, for example, competitively inhibits α-KG-dependent dioxygenases, leading to hypermethylation of DNA and histones at pro-inflammatory gene promoters, effectively silencing chronic inflammatory cascades.^28,78^ Concurrently, MQD-induced Nrf2 activation can shift the metabolic-epigenetic axis toward a permissive chromatin state at regulatory loci. This promotes sustained histone H3 lysine acetylation (H3K9ac and H3K27ac) at the promoters of anti-inflammatory genes such as IL-10 and FoxP3, thereby stabilizing the regulatory phenotypes of macrophages and Tregs. Understanding this metabolic-epigenetic coupling provides a profound mechanistic explanation for how MQDs achieve persistent, non-toxic immunotolerance, elevating our conceptual model from transient signaling interference to the permanent epigenetic reprogramming of dysregulated immune microenvironments.

Although current MQD studies primarily rely on transcriptional and metabolic readouts, direct validation of epigenetic remodeling will require integrative multi-omics approaches. Future investigations should combine ChIP-seq mapping of histone modifications (*e.g.*, H3K27ac and H3K9ac), DNA methylation profiling, ATAC-seq chromatin accessibility analysis, and transcriptomics to establish causal links between MQD-induced metabolic perturbations and immune-cell epigenetic reprogramming. Recent studies on engineered heterostructures have demonstrated how interfacial charge-transfer processes can be deciphered through complementary multi-level analyses, highlighting the value of systems-scale validation strategies for correlating material properties with downstream biological responses.^87^

## 4. Immunomodulatory performance of MQDs in inflammasome and regeneration

### 4.1. Direct T-cell modulation and stem cell-compatible immunoregulation

The first experimental evidence positioning MQDs as active immunomodulators in regenerative medicine was reported by Rafieerad *et al.*, who demonstrated that Ti_3_C_2_ MQDs exert selective control over adaptive immune activation while maintaining cytocompatibility with regenerative cell populations. In stimulated human lymphocyte cultures, MQDs significantly reduced the proportion of activated CD4^+^IFN-γ^+^ T cells from 87.1 ± 2.0% in controls to 68.3 ± 5.4%, indicating attenuation of Th1-type inflammatory signaling. Concomitantly, the percentage of immunosuppressive CD4^+^CD25^+^FoxP3^+^ regulatory T cells increased from 5.5 ± 0.7% to 8.5 ± 0.8%, suggesting an active shift toward immune tolerance rather than generalized immunosuppression.^79^

Importantly, this modulation occurred without compromising the viability of bone marrow-derived mesenchymal stem cells or induced pluripotent stem cell-derived fibroblasts. The absence of cytotoxic interference with stem cell populations addresses a major translational barrier in regenerative immunology, where biomaterial-driven inflammasome often limits therapeutic efficacy. Beyond immunophenotyping, MQDs were incorporated into a thermosensitive chitosan-based hydrogel, producing a conductive, injectable 3D platform suitable for cell delivery. Enhanced electrical conductivity, achieved without loss of injectability, may influence cellular communication within regenerative niches.

Conceptually, this study established three critical pillars for MQD-mediated immunomodulation: selective T-cell regulation, stem-cell compatibility, and functional integration into biomaterial scaffolds. Rather than acting as passive carriers, MQDs demonstrated intrinsic immunological activity capable of rebalancing inflammatory responses during tissue repair. These findings positioned MQDs as dual-function entities—simultaneously structural and immunologically instructive—within regenerative platforms.

### 4.2. Endothelial reprogramming and allogeneic immune suppression in transplant vasculopathy

Extending immunomodulatory exploration into transplantation pathology, Rafieerad *et al.* engineered tantalum carbide (Ta_4_C_3_T_*x*_) MQDs to address early allograft vasculopathy, a condition driven by endothelial activation and alloimmune T-cell responses. Unlike purely anti-proliferative strategies, this work targeted immune-endothelial crosstalk as a primary therapeutic axis. Ta_4_C_3_T_*x*_ MQDs were synthesized using a hydrofluoric acid-free method, yielding stable colloids enriched with bioactive TaO_2_ and Ta_2_O_5_ surface species. *In vitro* analyses revealed spontaneous uptake of MQDs by antigen-presenting endothelial cells, followed by modulation of surface receptor expression that reduced their capacity to activate allogeneic T lymphocytes. This finding is mechanistically distinct from direct T-cell suppression; instead, it demonstrates upstream immune regulation *via* alteration of antigen-presenting cell behavior. By attenuating endothelial immunogenicity, MQDs effectively interrupted the initiation phase of alloimmune activation.^80^

*In vivo* application further validated this approach. Administration of Ta_4_C_3_T_*x*_ MQDs ameliorated structural and cellular hallmarks of early transplant vasculopathy, including vascular thickening and inflammatory infiltration. The incorporation of tantalum—a metal with known anti-inflammatory and antiapoptotic characteristics—introduced compositional immunoengineering as a strategy to enhance biosafety and therapeutic durability. This study is significant for two reasons. First, it confirms that MQD-mediated immunomodulation extends beyond lymphocyte cultures into complex vascular inflammatory models. Second, it demonstrates that compositional tuning of MQDs can tailor immunological outcomes, supporting the concept of precision nano-immunomodulation in inflammatory diseases.

**Fig. 7:**
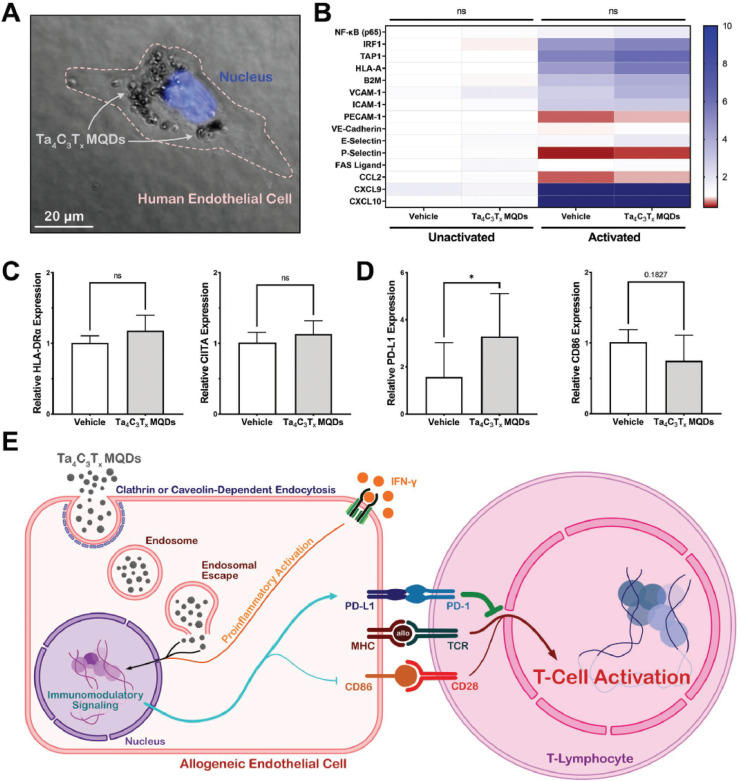
Mechanism of Ta_4_C_3_T_*x*_ MQD-induced endothelial reprogramming. (A) Intracellular localization of MQDs in HUVECs. (B and C) qPCR analysis of immunological gene pathways. (D) Quantitative shift in PD-L1 (co-inhibitor) and CD86 (co-activator) expression levels. (E) Schematic of the MQD-mediated immunomodulatory signaling axis. Reproduced from ref. 80 under the Creative Commons Attribution (CC BY) License. © 2021 Wiley-VCH GmbH.

The mechanistic foundation of this endothelial reprogramming lies in the precise interaction between MQDs and the vascular interface. As illustrated in Fig. 7A, Ta_4_C_3_T_*x*_ MQDs undergo rapid cellular internalization, localizing near the nucleus—a process facilitated by their rich surface functionalization and pH-triggered endosomal escape. Interestingly, genomic profiling *via* qPCR (Fig. 7B and C) reveals that these MQDs do not induce broad suppression of adhesion molecules or chemokines; instead, they execute a targeted ‘checkpoint switch.’ This is evidenced by a significant 3.3-fold upregulation of the T-cell co-inhibitor PD-L1 and a simultaneous downregulation trend of the co-activator CD86 on the HUVEC surface (Fig. 7D). By strategically altering this co-stimulatory ratio, MQDs transform the endothelium from a pro-inflammatory antigen-presenting surface into an immunotolerant landscape. This MQD-driven signaling cascade, summarized in Fig. 6E, effectively interrupts the crosstalk between the endothelium and alloreactive T-lymphocytes, providing a robust molecular basis for preventing transplant vasculopathy.

### 4.3. Spatial immune reprogramming within the tumor microenvironment

Ceylan *et al.* advanced the field by applying spatial transcriptomics to dissect *in situ* immune alterations following MQD administration in an orthotopic breast cancer model. Ti_3_C_2_T_*x*_ MQDs (≤10 nm) were tracked *via* intrinsic red fluorescence, enabling correlation between nanoparticle accumulation and localized immune gene expression profiles. Rather than uniform distribution, MQDs exhibited heterogeneous intratumoral localization, generating spatially distinct immune responses. Regions with high MQD accumulation demonstrated a tumor-suppressive transcriptional phenotype compared to low-accumulation regions or untreated tumors. Transcriptomic deconvolution identified recruitment and activation of B cells and induction of neutrophil degranulation and NETosis. These findings expand the immunological landscape of MQD activity beyond macrophage-centric paradigms, highlighting adaptive and innate immune cell involvement.^81^

The observation of B-cell activation suggests potential modulation of humoral immunity, while neutrophil extracellular trap (NET) formation indicates engagement of acute inflammatory effector mechanisms. Importantly, spatial omics analysis revealed that immune modulation was context-dependent and microenvironmentally structured, emphasizing that MQD effects cannot be interpreted solely through bulk tissue analysis. This study provides high-resolution molecular evidence that MQDs influence immune architecture within complex tissues. It underscores the importance of spatial biology in evaluating nanomaterial-driven immunomodulation and supports the hypothesis that MQDs can reshape immune cell distribution and signaling networks *in vivo*.

Beyond MQDs, studies on diverse nanomaterial systems have similarly demonstrated that biological activity is strongly influenced by nanoscale structural parameters, including surface functionality, electronic distribution, and interfacial charge-transfer characteristics. These observations support a broader structure–activity paradigm in nano–bio interactions, whereby subtle modifications in surface chemistry can reshape local biological responses. Recent structure-guided analyses of engineered porous nanomaterials have further highlighted the importance of correlating physicochemical descriptors with functional outcomes through integrated computational and experimental approaches.^88^ Such concepts may provide a valuable framework for interpreting the spatially heterogeneous immune responses observed following MQD accumulation within tumor tissues.

### 4.4. Dual-function regenerative platforms preventing tumor recurrence

Alkaya *et al.* integrated MQDs into 3D bioprinted hydrogel scaffolds designed for post-mastectomy breast reconstruction. The platform combined regenerative architecture with immunologically active nanomaterials to achieve simultaneous tumor suppression and tissue repair. Four scaffold variants—cellular, acellular, cellular + MQD, and acellular + MQD—were evaluated *in vivo* following tumor resection. Remarkably, the acellular MQD scaffold group exhibited no tumor recurrence by day 14 and demonstrated superior tissue regeneration based on histological and immunostaining analyses. The absence of recurrent tumor growth in the MQD-containing scaffold suggests that MQDs contributed to a local microenvironment unfavorable to malignant resurgence while supporting reparative processes.^82^

This dual-action outcome addresses a critical clinical challenge: balancing oncologic safety with reconstructive success. Conventional biomaterials may inadvertently create permissive niches for residual cancer cells. The incorporation of MQDs appears to alter local biological signaling in a manner that suppresses tumor regrowth without impairing tissue regeneration. The study highlights translational feasibility, demonstrating that MQD integration into advanced bioprinted constructs can produce functional outcomes *in vivo*. By bridging nanotechnology, tissue engineering, and immunological control, this work illustrates the potential of MQDs to serve as active modulators within regenerative platforms rather than passive structural additives.

**Fig. 8:**
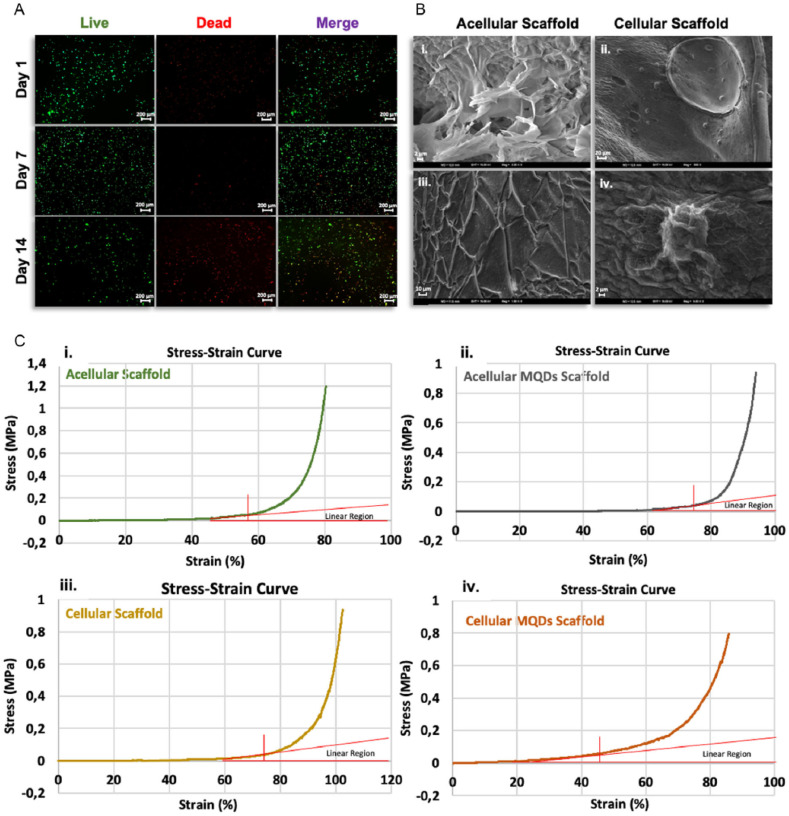
Characterization of 3D bioprinted hydrogel scaffolds: (A) live/dead fluorescence imaging of ASCs, (B) SEM micrographs showing scaffold morphology and cell distribution, and (C) stress–strain curves illustrating the mechanical properties of acellular and cellular scaffolds with and without MQDs. Reproduced with permission from ref. 82. © 2025 Wiley-VCH GmbH.

Panel (A) in Fig. 8 evaluates the cytocompatibility of MQD-integrated 3D bioprinted scaffolds, a critical prerequisite for their application as regenerative platforms following tumor resection. Live/dead fluorescence imaging demonstrates that low-dose MQD incorporation does not compromise adipose-derived stem cell (ASC) viability within the cellular scaffolds over time. The progressive increase in live cell density during early culture indicates cellular adaptation to the hydrogel microenvironment, while the absence of acute cytotoxic effects supports the use of MQDs as bioactive yet cell-friendly additives. This finding is particularly relevant for post-mastectomy reconstruction, where preservation of cellular function is essential for effective tissue regeneration.

Panel (B) provides structural insight into the printed scaffolds, revealing that MQD incorporation does not disrupt the intrinsic porous architecture required for nutrient diffusion and cell distribution. SEM images show comparable microstructural features between acellular and cellular scaffolds, with homogeneous ASC dispersion throughout the hydrogel matrix in cellular constructs. The maintenance of scaffold integrity despite MQD loading suggests that the nanodots function as active modulators of the local biological environment rather than as structural perturbants, enabling the scaffold to support both regenerative and anti-recurrence functions *in vivo*.

Panel (C) highlights the mechanical performance of the 3D-printed scaffolds, an essential factor for breast tissue engineering and long-term implantation. Stress–strain analysis indicates that MQD-containing scaffolds exhibit reduced stiffness and enhanced deformability compared to MQD-free counterparts, resulting in mechanical properties more closely aligned with native soft tissue. This moderated elasticity may facilitate improved tissue integration while avoiding the formation of rigid niches that could favor tumor recurrence. The cytocompatibility, preserved architecture, and tunable mechanics of MQD-integrated scaffolds provide a mechanistic basis for their observed ability to simultaneously support tissue regeneration and suppress malignant regrowth.

### 4.5. Redox activity and translational perspectives of MQD-based immunomodulatory nanosystems

The immunological implications of MQDs are closely linked to their redox capacity. Xiao *et al.* systematically characterized ROS generation by Ti_3_C_2_, Ti_2_C, and V_2_C MQDs under UV irradiation, identifying hydroxyl radicals, singlet oxygen, superoxide, and hydrogen peroxide *via* ESR spectroscopy and complementary assays. Their analyses attributed ROS production to unique band structures, efficient carrier separation, and enhanced electron transfer kinetics. From an immunological standpoint, controlled ROS generation can influence inflammatory signaling, antimicrobial responses, and tumor cell susceptibility. The ability of MQDs to generate multiple ROS species suggests versatility in modulating oxidative microenvironments. However, ROS magnitude and irradiation conditions are critical determinants of safety and efficacy, underscoring the need for context-specific optimization.^83^

Complementing experimental findings, Mousavi *et al.* reviewed advancements in MQD-based immunomodulatory cancer platforms, emphasizing biosafety, biodistribution, histopathology, and long-term toxicity concerns. They highlighted the necessity for scalable synthesis, advanced functionalization strategies, and rigorous cytotoxicity evaluation to enable clinical translation. The review situates MQDs within a broader framework of smart nanosystems designed for immune regulation and cancer therapy.^84^ Collectively, these studies define the translational trajectory of MQDs: redox-active, immunomodulatory nanoplatforms with demonstrated *in vivo* efficacy yet requiring systematic safety profiling and engineering refinement before widespread clinical adoption.^85^

Table 2 synthesizes representative experimental studies showing that MQDs act as active immunological regulators rather than passive nanomaterials. Across diverse disease models, MQDs modulate immunity at multiple levels, including adaptive T-cell polarization, endothelial antigen presentation, spatially organized immune responses in the tumor microenvironment, and redox-sensitive signaling linked to ROS generation. Importantly, these effects appear selective and context-dependent, enabling attenuation of pathological inflammasome while maintaining compatibility with regenerative cells and biomaterial scaffolds. The diversity in MQD composition (*e.g.*, Ti- *vs.* Ta-based systems and variable surface terminations) further suggests that elemental and surface engineering can tune immune outcomes, highlighting actionable design principles for immunoengineering. Collectively, the evidence positions MQDs as a versatile, mechanism-informed platform with translational potential in regenerative medicine, oncology, and transplantation, while also underscoring unresolved issues such as long-term biodistribution, degradation, and standardized safety evaluation.

**Table 2:** Comprehensive cross-sectional comparison of MQDs across contexts

MQD composition & properties	Model system & target	Core mechanism of efficacy	Translational relevance	Limitations/unresolved issues	Ref.
Ti_3_C_2_ MQDs	*In vitro* human lymphocytes & MSCs; *in vivo* chitosan hydrogel scaffold	T-cell polarization shift: selective down-regulation of pro-inflammatory Th1 pathway (↓ CD4^+^IFN-γ^+^) and promotion of CD4^+^CD25^+^FoxP3^+^ Tregs	Regenerative immunology & injectable stem cell delivery	Long-term biocompatibility and retention kinetics of MQDs within the hydrogel matrix remain unevaluated	79
• Ultrasmall (<10 nm)					
• High conductivity & hydrophilicity					
Ta_4_C_3_T_*x*_ MQDs	*In vitro* endothelial cells; *in vivo* murine transplant model	Upstream immuno-endothelial reprogramming: intracellular uptake, downregulation of co-stimulatory surface receptors, and prevention of allogeneic T-lymphocyte activation	Allograft vasculopathy & anti-transplant rejection	Tantalum-based MQDs face scalability issues in production and lack comprehensive long-term histopathology studies	80
• HF-free green synthesis					
• TaO_2_ and Ta_2_O_5_-enriched surface					
Ti_3_C_2_T_*x*_ MQDs	*In vivo* orthotopic breast cancer mouse model	Spatial microenvironment remodeling: local recruitment/activation of B cells, and activation of innate effector mechanisms (neutrophil degranulation and NETosis)	Tumor microenvironment (TME) modulation & bio-imaging	High spatial heterogeneity in tumor accumulation; precise molecular triggers for neutrophil activation remain unclear	[81]
• ≤10 nm diameter					
• Intrinsic red-emitting fluorescence					
Ti_3_C_2_ MQDs	*In vivo* post-mastectomy mouse model	Dual-action niche modulation: creation of a local immune-microenvironment unfavorable to malignant resurgence while maintaining porous architecture for ASC growth	Onco-regenerative scaffolds for post-mastectomy reconstruction	Long-term degradation rate of MQDs in bioprinted constructs must be calibrated to match tissue regeneration rates	[82]
• Embedded in 3D bioprinted hydrogel					
Ti_3_C_2_, Ti_2_C, V_2_C MQDs	Acellular ROS generation assays	Redox-active electron transfer: unique energy band structure and high carrier separation efficiency under UV (365 nm) light, generating multiple ROS species (˙OH, ^1^O_2_, O_2_˙^−^, H_2_O_2_)	Photodynamic therapy & redox-based antimicrobial/anticancer control	High reliance on external UV irradiation, which has limited tissue penetration depth for deep-seated clinical targets	[83]
• Hydrothermal synthesis					
• Variable bandgap structures					
Various MQD systems	*In vitro* & *in vivo* comprehensive literature	Systemic immunological crosstalk: modulation of bio-nano interfaces, surface charge interactions, and cellular uptake pathways across different organ systems	Clinical translation roadmap & biosafety standards	Significant gaps in standardized *in vivo* nanotoxicology, pharmacokinetics, and long-term biodistribution profiling	[84]
• Diversified compositions/surface functionalizations					

### 4.6. Design principles for precision immunomodulation

Beyond individual experimental outcomes, the collective body of evidence surrounding MQDs reveals an emerging framework of design principles that govern their immunological performance. Rather than functioning as passive nanocarriers, MQDs operate as active modulators whose biological effects are highly context-dependent and tunable through physicochemical engineering. Extracting these principles is essential for transitioning from empirical observations toward predictive nano-immunology.

First, immune modulation by MQDs appears to be threshold-dependent rather than binary. Studies demonstrate that MQDs do not indiscriminately suppress immune responses; instead, they recalibrate activation intensity. For example, selective reduction of pro-inflammatory T-cell subsets without broad cytotoxicity suggests a graded modulation profile. This implies that MQDs may function within an “immunological window” where dose, size, and surface state determine whether immune signaling is dampened, rebalanced, or locally activated.^79,83^ Such threshold behavior aligns with redox-sensitive immune checkpoints and underscores the need for quantitative immune profiling in future designs.

Second, compositional engineering directly shapes immunological bias. The incorporation of tantalum in Ta_4_C_3_T_*x*_ MQDs illustrates how elemental selection can confer intrinsic anti-inflammatory and antiapoptotic tendencies. This indicates that transition metal identity is not merely structural but immunologically instructive. Periodic table-guided material selection may therefore become a rational strategy for tailoring immune outcomes. Different metal cores may preferentially influence endothelial activation, lymphocyte proliferation, or inflammatory cytokine networks.

Third, spatial distribution critically determines immune consequence. Spatial transcriptomic mapping revealed that heterogeneous MQD accumulation produces region-specific immune phenotypes within tumors. This finding establishes distribution heterogeneity as a functional variable rather than a pharmacokinetic limitation.^80,84^ Future immunomodulatory platforms must therefore consider spatial deposition patterns as part of therapeutic design, particularly in tissues characterized by structural complexity.

Fourth, integration within biomaterial scaffolds amplifies immunological impact. MQD-containing hydrogels and bioprinted constructs demonstrate that immune modulation is enhanced when nanomaterials are embedded within structural matrices. This suggests a cooperative interaction between physical architecture and immunological signaling. Scaffold conductivity, mechanical stability, and retention kinetics may synergize with MQD-driven cellular effects, indicating that nano–macro integration is a key translational axis. Finally, ROS-generating capability provides a controllable biochemical lever. The ability of MQDs to generate multiple ROS under defined irradiation conditions offers an externally adjustable immunomodulatory tool. However, the therapeutic balance between pro-inflammatory oxidative bursts and controlled redox signaling must be precisely calibrated. This reinforces the concept that MQDs operate as redox-tunable immunomodulators rather than fixed-function agents.^80,83^

Collectively, these principles define a conceptual shift: MQDs should be engineered not solely for delivery efficiency or cytotoxic potency, but for programmable immune interaction. Dose optimization, elemental composition, spatial targeting, scaffold integration, and redox tuning form a multidimensional design matrix. Establishing standardized immunological evaluation platforms—incorporating phenotypic, transcriptomic, and functional assays—will be essential to translate MQD systems into precision nano-immunotherapies for inflammatory diseases and regenerative medicine. This principle-driven perspective elevates MQDs from promising nanomaterials to a modular platform technology capable of rational immune system engagement.

### 4.7. Systems-level immunology and mechanistic cross-talk: beyond basic immunosuppression

To move beyond a descriptive catalog of empirical observations, it is critical to conceptualize how MQDs function not as simple, localized immunosuppressants, but as dynamic, systems-level orchestrators of immune homeostasis. Pristine nanomaterials often trigger binary cellular responses—either inducing robust inflammasome (foreign body response) or enacting generalized, systemic immunosuppression that compromises host defense. In contrast, the biological performance of MQDs across inflammatory, oncologic, and regenerative contexts points toward a sophisticated, multi-system immuno-tactical paradigm. By engaging both innate and adaptive pathways simultaneously, MQDs facilitate a network-level recalibration of the tissue microenvironment. The transition-metal core and tunable surface terminations of MQDs allow them to act as heterogeneous catalysts at the nano–bio interface, modulating intracellular signaling cascades in a non-linear, threshold-dependent manner.^79,80^ Instead of shutting down immune pathways, MQDs tune the activity of master transcription factors (*e.g.*, NF-κB, Nrf2) to shift macrophage phenotypes while cooperatively guiding T-cell polarization toward regulatory phenotypes.

However, to elevate this field from empirical screening to predictable clinical application, several critical knowledge gaps must be addressed. Current literature relies heavily on static, phenotypic endpoint markers (such as surface receptor expression or isolated cytokine secretions), which fail to capture the highly dynamic, temporal trajectory of immune remodeling. Future studies must employ high-resolution, multi-omics approaches—including single-cell RNA sequencing (scRNA-seq), spatial transcriptomics, and deep immunoproteomics—to map the kinetic evolution of the MQD-induced immune response. This systemic resolution is particularly urgent for understanding the “onco-regenerative paradox,” where MQDs must concurrently suppress malignant cells while promoting stem cell-mediated tissue repair within the same microenvironment.^81,82^ Only by unraveling these complex biological feedback loops and mapping the epigenetic rewiring of immune cells can we fully exploit the structural versatility of MQDs, transforming them from basic nanostructures into programmable, clinically viable immunotherapeutic platforms.

## 5. Biosafety, redox risks, and clinical translation pathways

### 5.1. Pharmacokinetics, biodistribution, and metabolic fate

A fundamental requirement for the clinical translation of MQDs is the thorough elucidation of their *in vivo* pharmacokinetic profiles, systemic biodistribution, and long-term metabolic fates. Upon systemic administration, these transition metal-based nanomaterials immediately interact with biological fluids, leading to the rapid formation of a “protein corona” that alters their synthetic identity and dictates cellular internalization pathways. Most MQDs are heavily sequestered by the organs of the mononuclear phagocyte system (MPS), particularly the liver and spleen, where resident macrophages actively internalize them *via* clathrin- or caveolae-mediated endocytosis.

The structural integrity and subsequent clearance kinetics of MQDs are highly dependent on their lateral size, composition, and surface chemistry (such as –OH, –F, and –O terminations). Ultrasmall MQDs (<10 nm) theoretically fall below the renal filtration threshold, favoring rapid urinary clearance and reducing systemic exposure. However, surface-charge-mediated aggregation or interaction with plasma proteins can significantly increase their effective hydrodynamic diameter, shifting the elimination route toward hepatobiliary excretion.

A critical knowledge gap remains regarding the chemical transformation of these materials within lysosomal microenvironments. While titanium-based systems (Ti_3_C_2_) may undergo gradual oxidation into amorphous titanium dioxide (TiO_2_) and carbonaceous byproducts, heavier transition metal cores, such as tantalum (Ta_4_C_3_), exhibit much greater chemical stability, raising concerns over permanent tissue retention. Longitudinal studies extending over several months are essential to evaluate the risk of chronic, low-dose accumulation-induced toxicity. Without rigorous, real-time tracking of degradation kinetics using techniques like inductively coupled plasma mass spectrometry (ICP-MS) and radio-labeling, the safety of MQDs remains speculative, hindering regulatory approval and clinical translation.

### 5.2. The redox paradox: dual risks and immunogenicity

The intrinsic redox activity of MQDs represents a major therapeutic asset, yet it simultaneously introduces a profound safety paradox regarding biocompatibility. In oncology and antimicrobial therapies, the capacity of MQDs to generate diverse reactive oxygen species (ROS)—including hydroxyl radicals (˙OH), singlet oxygen (^1^O_2_), and superoxide anions (O_2_˙^−^) under external stimuli or cellular interactions—is exploited to destroy pathological targets. However, when applied within systemic or highly vascularized microenvironments, this potent oxidative capability poses a serious “dual risk” of collateral damage to healthy surrounding tissues.

Excessive intracellular ROS generation can trigger lipid peroxidation of mitochondrial and cell membranes, leading to cytochrome c leakage, DNA strand breaks, and ultimately, unintended necrotic or apoptotic cell death in healthy organs. Furthermore, the immunogenicity of MQDs—specifically their potential to act as haptens or stimulate sub-clinical, chronic inflammatory states—remains largely unexplored. Intracellular accumulation of redox-active nanodots can persistently stimulate the NLRP3 inflammasome pathway, prompting the sustained release of pro-inflammatory cytokines such as IL-1β and IL-18.

Additionally, the complex surface chemistry of MQDs may trigger the complement cascade *via* classical or alternative pathways, leading to transient hypersensitivity reactions. To safely navigate this redox paradox, researchers must define a strict “therapeutic window” where the antioxidant properties of MQDs (acting as free radical scavengers at low concentrations) can be safely decoupled from their pro-oxidant, cytotoxic behaviors. Developing smart, microenvironment-responsive MQDs that only exhibit redox activity under specific pathological conditions (*e.g.*, acidic tumor pH or high local GSH concentrations) represents a vital engineering strategy to mitigate systemic immunogenicity and oxidative side effects.

At present, a universally accepted therapeutic window for MQDs has not been established due to substantial variations in composition, surface chemistry, and experimental models. Nevertheless, future safety frameworks should quantitatively define concentration-dependent transitions between antioxidant and pro-oxidant behavior using standardized ROS-generation metrics, intracellular redox biomarkers, and dose–response profiling. Analogous studies in engineered heterojunction systems have demonstrated that advanced charge-transfer quantification techniques can accurately correlate carrier dynamics with reactive species production.^89^ Extending similar quantitative approaches to MQDs may facilitate the determination of safe exposure ranges, ROS thresholds, and clinically relevant dosing parameters required for translational development.

### 5.3. Limitations of preclinical models in predicting human safety

A major roadblock to the clinical translation of MQD-based therapeutics is the poor predictive power of current preclinical safety models. The vast majority of published studies rely on small rodent models (mice and rats) to evaluate acute toxicity and immunomodulatory efficacy. However, the murine immune system possesses fundamental physiological differences from the human counterpart, including distinct lymphocyte ratios, divergent toll-like receptor (TLR) expression patterns, and different nitric oxide production kinetics in macrophages. These biological discrepancies mean that a nanomaterial deemed “biocompatible” in healthy mice may trigger unexpected immunogenic reactions or complement-mediated toxicities when introduced into human patients.

Furthermore, standard preclinical assays typically focus on short-term, acute endpoints (*e.g.*, 777-to-141 414 day survival and basic organ histopathology), failing to simulate the chronic, multi-decade lifetime of human patients. Rodent models also fail to reflect the heterogeneous, comorbid physiological states of actual clinical candidates, who may suffer from pre-existing cardiovascular, renal, or immune dysfunction that alters nanoparticle pharmacokinetics and intensifies systemic toxicity.

Additionally, traditional *in vitro* colorimetric assays (such as MTT or LDH) frequently interact directly with MQDs due to their high optical absorbance and surface reactivity, resulting in false-positive or false-negative toxicity readings. To overcome these limitations, future research must shift toward advanced evaluation platforms. Incorporating humanized mouse models with reconstituted human immune systems, high-throughput microfluidic organ-on-a-chip technologies, and long-term primate safety profiles is essential. Standardizing these human-centric, multi-dimensional screening methodologies represents the only viable pathway to ensure that MQD systems can transition safely from the laboratory bench to the patient's bedside.

## 6. Conclusion and outlook: toward precision immunomodulation and translational MQD engineering

MQDs have emerged as a distinct class of redox-active nanomaterials capable of modulating immune responses across inflammatory, oncologic, and regenerative contexts. Experimental evidence demonstrates that MQDs do not function merely as passive carriers but actively reshape immune landscapes by regulating T-cell activation, influencing endothelial immunogenicity, altering tumor-associated immune populations, and integrating within regenerative scaffolds. Their physicochemical tunability—including compositional engineering, surface functionalization, and redox adaptability—enables context-dependent immune rebalancing rather than indiscriminate suppression. Importantly, MQDs exhibit a dual capacity: attenuating pathological inflammasome while preserving or enhancing regenerative processes, addressing a long-standing challenge in biomaterials science where immune activation often compromises tissue repair.

Looking ahead, the concept of precision immunomodulation offers an important direction for MQD development. By leveraging their optical and electrical properties, MQDs may enable spatiotemporally controlled immune intervention, such as light-triggered activation, controllable release of bioactive cues, or electrically responsive modulation of cell signaling within target tissues. Such programmable behavior could expand the therapeutic scope of MQDs from passive nanomaterials to active immunoengineering platforms.

In parallel, integrating MQDs with other therapeutic modalities may further enhance clinical value. Their use as adjuncts in immunotherapy, cell therapy, and gene therapy may provide synergistic effects by improving local immune control, enhancing delivery efficiency, or creating favorable microenvironments for therapeutic cells and biomolecules. These combination strategies may be especially relevant in complex diseases requiring coordinated immune and tissue-level interventions.

Another promising direction is computational and AI-driven design. Machine learning models could help predict relationships between MQD structure, surface chemistry, and immune outcomes, accelerating material discovery and reducing trial-and-error synthesis. Data-driven approaches may also support the identification of optimal design rules for balancing efficacy, selectivity, and biosafety.

A particularly attractive future scenario is the development of predictive MQD immunology platforms. For example, machine-learning models trained on physicochemical descriptors—including MQD size, surface terminations (−OH, –O, –F, and –NH_2_), zeta potential, and transition-metal composition—could be used to predict downstream immune phenotypes such as macrophage M1/M2 polarization, Treg induction, cytokine secretion profiles, or endothelial immunogenicity. Such a framework would transform MQD development from empirical screening into a design-driven process, where desired immunological outcomes are specified prior to synthesis. Similar structure-guided material optimization strategies have successfully linked compositional parameters to functional performance in advanced nanomaterial systems,^91^ highlighting the feasibility of predictive immunoengineering approaches.

Finally, future studies should increasingly rely on advanced characterization techniques, including spatial transcriptomics, single-cell sequencing, and other high-resolution omics platforms, to decode the tissue-level immune remodeling induced by MQDs. These methods will be essential for capturing the spatial and cellular heterogeneity of MQD responses and for defining mechanistic signatures of immunomodulation *in vivo*. Collectively, MQDs represent a modular and highly tunable platform for rational immune system engagement. With continued advances in precision design, multimodal therapy integration, computational modeling, and high-resolution biological interrogation, MQDs hold substantial promise for next-generation immunomedicine and regenerative applications.

## Conflicts of interest

The authors declare that they have no known competing financial interests or personal relationships that could have appeared to influence the work reported in this paper.

## Data Availability

No primary research results, software or code have been included and no new data were generated or analysed as part of this review.
